# Clinical impact and outcome of clostridium non-difficile infection in critically ill patients

**DOI:** 10.1186/2197-425X-3-S1-A117

**Published:** 2015-10-01

**Authors:** K Rutter, S Walther, T Horvatits, A Drolz, K Roedl, C Bopp, R Wüstenberg, S Kluge, V Fuhrmann

**Affiliations:** Department of Intensive Care Medicine, University Medical Center Hamburg-Eppendorf, Hamburg, Germany

## Introduction

Clostridium species are gram-positive, anaerobic, spore-forming bacteria and some species have pathogenic nature. There is limited data regarding Clostridium non-difficile infection in critically ill patients available. Symptoms of infection are often non-specific, which leads to delayed diagnosis and therapy initiation in these patients.

## Objectives

Aim of this study was to evaluate patients with positive microbiological cultures of Clostridium non-difficile infection with the need of critical care medicine.

## Methods

Patients with microbiological result of Clostridium non- difficile infection admitted to the intensive care unit (ICU) were included in this study. Patient´s characteristics including admission diagnosis, severity of illness (SOFA- score), therapeutic procedures and outcome were recorded.

## Results

A total of 47 critically ill patients (32 men, mean age 66 ± 9 years, mean SOFA-score on admission 9 ± 3) with Clostridium non-difficile infection were included in this study. The most common pathogens were Clostridium innocum (n = 23), Clostridium perfringens (n = 11), Clostridium tertium (n = 6) and others (n = 7). Pathogens were detected in 62% intra-abdominal, in 29% in blood cultures and 8% of patients had soft tissue infection. Intra-abdominal infections (71%) were the most common source of infection.

Admission diagnoses were septic shock (54%), surgical treatment (44%) and others (2%). Highest incidence of septic shock was seen in patients with Clostridium innocum infection, (p < 0.05). Preexisting, mostly abdominal (90%) malignancy was seen in 46% of these patients. Invasive ventilation was needed in 50%, vasopressor therapy in 71% and renal replacement therapy in 38%. The overall ICU mortality was 45%. Highest ICU mortality rate was found in patients with Clostridium innocum infection (45%), followed by Clostridium perfringens (23%) and Clostridium tertium (17%). Patients with septic shock showed significantly higher mortality rates (p < 0.05). By means of source of infection highest mortality rate was found in abdominal infection (65%) followed by bacteremia (43%).

## Conclusions

Clostridium non-difficile infection in critically ill patients is associated with high mortality and organ failure. Worst outcome was observed in patients with septic shock.

